# What is the prevalence of peri-implantitis? A systematic review and meta-analysis

**DOI:** 10.1186/s12903-022-02493-8

**Published:** 2022-10-19

**Authors:** Pedro Diaz, Esther Gonzalo, Luis J. Gil Villagra, Barbara Miegimolle, Maria J. Suarez

**Affiliations:** Department of Conservative Dentistry and Bucofacial Prosthesis, Faculty of Odontology, University Complutense of Madrid (UCM), Pza Ramón y Cajal S/N, 28040 Madrid, Spain

**Keywords:** Dental implants, Peri-implantitis, Epidemiology

## Abstract

**Background:**

Peri-implantitis is a usual finding but estimates of its prevalence fluctuate very much. This may be due to the wide variety of disease definitions. This systematic review aims to estimate the overall prevalence of peri-implantitis and the effect of different study designs, function times and use of probing depth on prevalence rate.

**Methods:**

Following electronic and manual searches of the literature published from January 2005 to December 2021, data were extracted from the studies fitting the study criteria. Fifty-seven articles were included in this study.

**Results:**

Prevalence of peri-implantitis was 19.53% (95% CI 12.87–26.19) at the patient-level, and 12.53% (95% CI 11.67–13.39) at the implant-level and it remains highly variable even following restriction to the clinical case definition. The use of probing depth like diagnostic criteria affected the prevalence data.

**Conclusion:**

The results indicate that it remains essential the identification of the diagnostic markers for more accurate disease classification.

## Background

Dental implants are currently one of the safest alternatives for the replacement of missing teeth, regardless of their cause. This treatment has shown a high degree of predictability, with a survival rate in the range of 90–95% for more than 5 years [1].

It is important to discriminate between survival and success rates of treatment. An implant with enough insertion and no mobility (positive survival) can be a failure (negative success) if it exhibits any coil or constant inflammation of the peri-implant soft tissue. The incidence of technical and biological complications appears to be common [2–4], and these complications can have substantial economic implications and effects on the perception of treatment of the patient [5–8]. As the number of patients receiving dental implants is continually growing, the prevention and treatment of associated complications represents a serious and relevant challenge.

Within the biological complications, peri-implant diseases are considered the most relevant. They have an infectious cause and two entities have been described: mucositis and peri-implantitis [9]. Peri-implantitis is characterized by a destructive inflammatory lesion of polymicrobial etiology that affects both soft and hard tissues leading to progressive peri-implant bone loss, along with the formation of a pocket and inflammation in peri-implant tissues [2, 10]. Thus, the pathognomonic clinical sign of peri-implantitis will be the increase in pocket depth accompanied by bleeding and sometimes suppuration [11].

In order to better understand the magnitude of peri-implant diseases, it is mandatory to understand their epidemiology. It has therefore been suggested that epidemiological studies with a cross-sectional design, adequate sample sizes, and clinical and radiographic records are necessary to study the prevalence and risk indicators of peri-implant diseases [12]. Previous study reported that the prevalence of peri-implantitis ranged from 14.38 to 24.27% [13]. The reported variability may depend on different factors, including the follow-up period or disease definition. The definition is quite controversial and many different definitions have been proposed [14], until the 2017 World Workshop on the Classification of Periodontal and Peri**‐**Implant Diseases and Conditions [15] proposed a new classification of periodontal and peri-implant diseases, where, in the absence of a previous examination, the diagnosis of peri-implantitis may be based on the combination of bleeding on probing (BOP) and/or suppuration, probing depth ≥ 6 mm, and loss of supporting bone ≥ 3 mm. Another relevant factor is the use of convenience samples instead of randomized samples, which ultimately results in a potential selection bias [16].

The variability in the prevalence of peri-implantitis can be also explained by the different clinical parameters used to define the disease in the different studies, especially in terms of the magnitude of loss of supporting bone and the probing depth, the heterogeneity of the groups evaluated, or the individual risk factors of each population. Individual risk factors significantly increase the prevalence of peri-implantitis and may include the patient's previous history of periodontal disease, smoking habit, poor oral hygiene, diabetes and genetic factors [17].

Due to the great heterogeneity in peri-implantitis prevalence data, it is necessary to evaluate the currently data to approach the knowledge of its epidemiology and provide the clinicians relevant information to evaluate, for example, new complementary therapies to mechanical debridement treatment, such as probiotic or postbiotic gels [18, 19]. Therefore, the aim of this systematic review is to estimate the prevalence of peri-implantitis and its variations according to the applied definition and the elapsed time.

## Methods

The present study was registered in PROSPERO with ID CRD42022313472. The practice-oriented research question was: “What is the current state of knowledge regarding the prevalence of peri-implantitis in patients treated with titanium dental implants?”.

### Search strategy and search terms

A thorough search for literature was conducted from 1 December 2005 to 31 December 2021, using the following electronic databases: MEDLINE/PubMed, Web of Science, Science Direct and the Cochrane Library. The main key search terms used, alone or in combination with Boolean operators, for different searches were: "dental implants", "peri-implantitis" and "epidemiology”. The first combination was “dental implants and peri-implantitis” and the second option was “peri-implantitis epidemiology”. This search strategy was adapted for use in the various databases. Table 1 shows the results.

**Table 1:** Number of articles found according to search strategy

Database	“Dental implants” + “peri-implantitis”	“Peri-implantitis” + “epidemiology”	Total
PubMed	681	53	734
Web Of Science	764	56	820
Cochrane	1054	19	1073
Science Direct	1241	68	1309
TOTAL	3740	196	3936

### Screening and selection: eligibility criteria

The inclusion criteria were as follow: original studies describing the diagnosis of peri-implantitis (BOP, probing depth, loss of supporting bone); observational and experimental studies (cross-sectional, longitudinal, cohort or randomized controlled trial) with original prevalence data published over the past 16 years; studies published in peer review system journals; articles published in English language; and technical possibility to access the full text. The exclusion criteria were: studies where the number of subjects treated with implants were less than 10; or if the minimum time of function of implants was less than 5 years; and studies including subjects with clotting disorders.

The titles and summaries identified in the initial search were evaluated by three authors (PD, LJG-V and EG) for eligibility after removing duplicate items. Studies that appeared to meet the inclusion criteria were recovered in their full-text version and evaluated. A manual search of additional relevant titles was also carried out in the references section of each article. Any disagreement among the reviewers was resolved by discussion with all authors until consensus was reached.

### Quality assessment of the risk of bias

The Cochrane Collaboration tool for assessing risk of bias was applied to the pre-selected papers [20]. Articles with ‘high risk’ were rejected.

### Data extraction and collection

Once the articles meeting the inclusion criteria were identified, the following data for each article was collected using a specific form: surname and first author's name, geographic scope, sample size type of study design, type of peri-implantitis diagnosis, year and type of publication, and data on peri-implantitis for the calculation of prevalence at both, the patient level (number of patients with peri-implantitis/total number of patients × 100) and the implant level (number of implants with peri-implantitis/total number of implants × 100). This information was felt in different sections. These tasks were performed by the same three authors (PD, LJG-V and EG).

### Statistical analysis

In order to reduce the heterogeneity of the results and to facilitate their interpretation, the studies were grouped according to diagnostic criteria into four groups: Group 1 (BOP, PD ≥ 6 mm and Loss of supporting Bone ≥ 3 mm), Group 2 (BOP, PD ≥ 6 mm and BL ≥ 2 mm), Group 3 (Progressive Bone Loss), and Group 4 (Other Criteria). A meta-analysis of Group 2 was performed due to its large specific weight (31 articles). The program used was MetaXL, tool for meta-analysis in Microsoft Excel for Windows. Sensitivity analyzes were performed, replicating the results after the exclusion of a study, to observe the robustness of the analysis and the influence of the eliminated study. Heterogeneity was evaluated using the I^2^ test which analyzes the proportion of total variability between studies explained by heterogeneity [21]. To prevent the presence of publication bias, we used the Egger´s regression test (p ≥ 0.1) complemented with Doi plot [22].

## Results

**Fig. 1:**
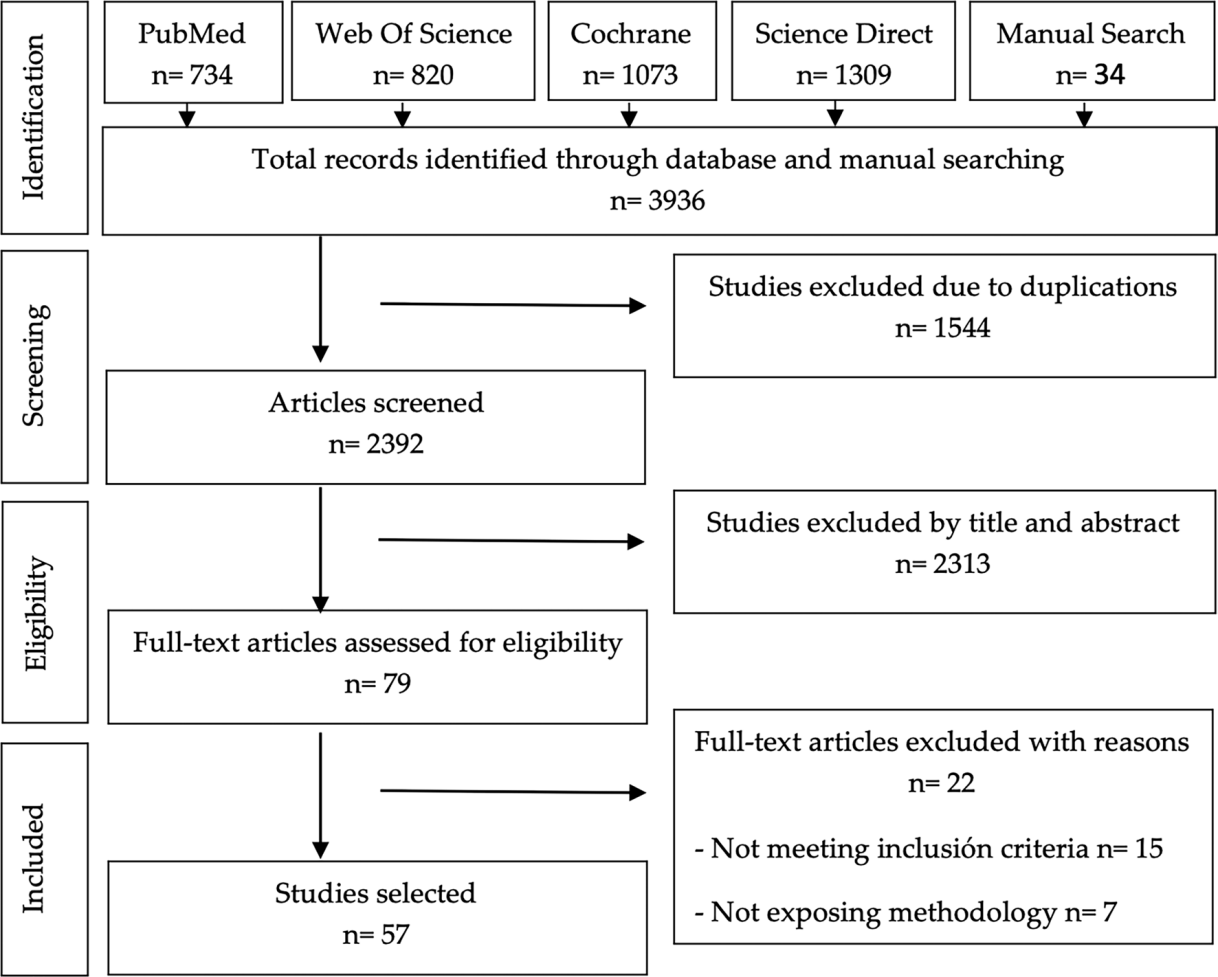
PRISMA flowchart

This initial electronic search produced 3902 articles and the manual search 34 articles. After eliminating duplication, examining, and applying inclusion criteria, 79 articles were included for data extraction and full-text evaluation. However, 22 articles were excluded because they did not meet the objectives of the review, or they did not have a clear methodology. Therefore, a total of 57 articles [23–79], were selected as relevant to the objectives of the review. The PRISMA flowchart in Fig. 1 synthesizes the screening and selection processes. The study designs were as follow: 18 cross-sectional studies, 18 longitudinal studies, 1 case–control study, 17 cohort studies, and 3 randomized controlled trials (Tables 2, 3, 4, 5, 6, 7, 8, 9). The study of Rodrigo et al. [66] was included in Groups 1 and 2.

**Table 2:** Characteristics of studies Group 1 (BOP + probing depth ≥ 6 mm + bone loss ≥ 3 mm)

Studies	Characteristics						Results	
	Population	Sample (N)(P)	Sample (N)(DI)	Design follow-up	Time load (Y)	Diagnostic	Rate (P) (%)	Rate (DI) (%)
Costa [29]	SA	80	221	Longitudinal	5 ± 0.5	BOP/suppuration + PD ≥ 5 mm + BL ≥ 3 mm	Missing	31.2217
Rodrigo [66]	EU	275	474	Cohort	9 ± 1.7	BOP + BL ≥ 3 mm	14.1818	11.3924
Rocuzzo (2012)	EU	101	228	Cohort	10	BOP + PD ≥ 6 mm + BL ≥ 3 mm	29.7029	17.1053
Rocuzzo (2014)	EU	123	246	Longitudinal	10	BOP + PD ≥ 6 mm + BL ≥ 3 mm	Missing	7.7236
Shimchuk [71]	USA	95	220	Cross-sectional	10.9	BOP/suppuration + PD ≥ 6 mm + BL ≥ 3 mm	6.3158	3.6363
Tenenbam (2017)	EU	52	108	Cohort	10.8 ± 1.7	BOP/suppuration + PD ≥ 5 mm + BL ≥ 4.5 mm	15.3846	12.037
Trullenke-Eriksson (2015)	EU	105	342	Longitudinal	13.19 ± 3.7	BOP/suppuration + PD ≥ 5 mm + BL > 3 mm	Missing	1.7544

**Table 3:** Characteristics of studies Group 2 (BOP + probing depth ≥ 6 mm + bone loss ≥ 2 mm)

Studies	Characteristics						Results	
	Population	Sample (N)(P)	Sample (N)(DI)	Design follow-up	Time load (Y)	Diagnostic	Rate (P) (%)	Rate (DI) (%)
Adler [23]	EU	376	1095	Cohort	11 (9–15)	BOP/suppuration + PD > 5 mm + BL ≥ 2 mm	21.0106	Missing
Ahn [25]	Korea	111	209	Longitudinal	> 7	BOP + PD > 5 mm + BL > 2 mm	Missing	16.7464
Bäumer [26]	EU	100	242	Longitudinal	10 ± 0.31 (9.5–10.7)	BOP/suppuration + BL > 2 mm	16	10.3306
Becker [27]	EU	92	328	Longitudinal	14 ± 1.9	BOP/suppuration + PD ≥ 5 mm + BL ≥ 2.5 mm	Missing	9.7561
Dalago [30]	SA	183	916	Cross-sectional	> 5	BOP/suppuration + PD > 5 mm + BL > 2 mm	16.3934	7.3144
Daubert [31]	USA	96	225	Cross-sectional	10.9 ± 1.5 (8.9–14.8)	BOP/suppuration + PD ≥ 4 mm + BL ≥ 2 mm	26.0417	16
Den Hartog [33]	EU	93	93	Randomized Controlled Trial	5	BOP/suppuration + BL ≥ 2 mm	15.0538	15.0538
Derks[11]	EU	427	1578	Cross-sectional	9	BOP/suppuration + BL > 2 mm	14.5199	7.9848
Fransson [36]	EU	182	1070	Cross-sectional	5 to 20	BOP/suppuration + PD > 6 mm + BL > 2 mm	Missing	39.1589

**Table 4:** Characteristics of studies Group 2 (BOP + probing depth ≥ 6 mm + bone loss ≥ 2 mm)

Studies	Characteristics						Results	
	Population	Sample (N)(P)	Sample (N)(DI)	Design follow-up	Time load (Y)	Diagnostic	Rate (P) (%)	Rate(DI) (%)
Gamper [38]	EU	56	143	Randomized Controlled Trial	5	BOP/suppuration + PD ≥ 5 mm + BL ≥ 2 mm	10.7143	7.6923
Gatti [39]	EU	56	227	Cohort	5	BOP/suppuration + PD > 5 mm + BL > 2 mm	3.5714	1.7621
Gonzalez-Glez (2020)	EU	65	558	Longitudinal	5	BOP/suppuration + PD ≥ 5 mm + BL > 2 mm	16.9231	1.9713
Guarneri (2018)	EU	74	166	Longitudinal	5	BOP/suppuration + PD > 5 mm + BL > 2 mm	13.5135	7.8313
Hu [42]	Singapore	200	284	Cohort	6.8	BOP/ + incr PD + BL > 2	13	10.2113
Ioannidis[43]	EU	64	103	Randomized Controlled Trial	5	BOP + BL > 2 mm	Missing	6.7961
Karlsson[44]	EU	596	Missing	Cohort	9	BOP/suppuration + BL > 2 mm	18.4564	Missing
Kosdsland (2010)	EU	104	295	Cross-sectional	8.4 + 4.6	BOP/suppuration + PD ≥ 4 mm + BL ≥ 2 mm	47.1154	36.6102
Konstantinidis [46]	EU	90	226	Cross-sectional	5.5	BOP + PD > 5 mm + BL > 2 mm	13.3333	6.1947

**Table 5:** Characteristics of studies Group 2 (BOP + probing depth ≥ 6 mm + bone loss ≥ 2 mm)

Studies	Characteristics						Results	
	Population	Sample (N)(P)	Sample (N)(DI)	Design follow-up	Time load (Y)	Diagnostic	Rate (P) (%)	Rate (DI) (%)
Lee [47]	Australia	60	117	Case–control	8 (5–13.46)	BOP + PD ≥ 5 mm + BL > 2 mm	26.6666	19.6581
Marrone [49]	EU	103	266	Cross-sectional	> 5	BOP/suppuration + PD > 5 mm + BL > 2 mm	36.8932	22.9323
Meijer [50]	EU	140	276	Cohort	5	BOP/suppuration + BL ≥ 2 mm	17.1428	11.5942
Nobre [53]	EU	353	1238	Cohort	5	BOP/suppuration + PD ≥ 5 mm + BL ≥ 2 mm	24.0793	Missing
Papaspyridakos (2019)	USA	41	359	Cohort	5	BOP/suppuration + BL > 2 mm	Missing	8.0779
Pimentel [56]	SA	147	490	Cross-sectional	> 5	BOP/suppuration + PD > 4 mm + BL > 2 mm	19.0476	9.1837
Ravald [57]	EU	46	371	Longitudinal	12 to 15	BOP/suppuration + PD ≥ 4 mm + BL ≥ 2 mm	21.7391	3.7786
Ravidá [58]	USA	145	382	Longitudinal	5.2–6.5	BOP + BL > 2 mm	16.5517	9.9476
Rodrigo [66]	EU	275	474	Cohort	9 ± 1.7	BOP + BL ≥ 2 mm	24	19.6202

**Table 6:** Characteristics of studies Group 2 (BOP + probing depth ≥ 6 mm + bone loss ≥ 2 mm)

Studies	Characteristics						Results	
	Population	Sample (N)(P)	Sample (N)(DI)	Design follow-up	Time load (Y)	Diagnostic	Rate (P) (%)	Rate (DI) (%)
Rokn [67]	Iran	134	478	Cross-sectional	5	BOP/suppuration + BL > 2 mm	20.1492	8.7866
Romandini (2020)	EU	99	458	Cross-sectional	7.8	BOP/suppuration + BL ≥ 2 mm	56.5656	27.9476
Tey [75]	Singapore	194	266	Longitudinal	5.2 ± 1.5	BOP + PD ≥ 6 mm + BL ≥ 2.5 mm	8.2474	7.1428
Vandeweghe [77]	EU	33	197	Longitudinal	14.3(10–21)	BOP/suppuration + PD > 6 mm + BL ≥ 2.5 mm	Missing	4.0609

**Table 7:** Characteristics of studies Group 3 (Progressive bone loss)

Studies	Characteristics						Results	
	Population	Sample (N)(P)	Sample (N)(DI)	Design follow-up	Time load (Y)	Diagnostic	Rate (P) (%)	Rate (DI) (%)
Chappuis [28]	EU	67	95	Cohort	20	BOP + infection + BL progressive	Missing	13.6842
Gurgel [32]	SA	155	Missing	Cross-sectional	5	BOP/supp + PD > 5 mm + BL Rx visibl	28.3871	Missing
Francetti [35]	EU	46	56	Longitudinal	5	BOP/supp + increPD + BL Rx visible	0	0
French [37]	EU-USA	2060	4591	Cohort	6–7	BOP/suppuration + PD > 2 mm + BL > 1 mm least year	11.6990	4.7048
Pandolfi [54]	EU	475	1991	Cohort	10	BOP/supp + BL changes	9.6842	12.09081
Ravidá[59]	USA	99	221	Cohort	10.6 ± 4.5	BOP/supp + increPD + BL progressiv	20.4	15
Rinke [61]	EU	89	Missing	Cross-sectional	5.5 ± 2	BOP/supp + PD ≥ 4 mm + BL progress	11.2359	Missing
Rinke[62]	EU	65	112	Longitudinal	6.8 + 1.96	BOP/supp + PD ≥ 5 mm + BL progress	9.2308	Missing
Rodrigo [65]	EU	22	68	Cohort	5	BOP/supp + PD ≥ 4 mm + BL significa	Missing	5.8823
Simonis [72]	EU	55	124	Longitudinal	10 to 16	BOP + PD ≥ 5 mm + BL > 0.2 mm/year	Missing	16.9355

**Table 8:** Characteristics of studies Group 3 (progressive bone loss)

Studies	Characteristics						Results	
	Population	Sample (N)(P)	Sample (N)(DI)	Design follow-up	Time load (Y)	Diagnostic	Rate (P) (%)	Rate (DI) (%)
Swierkot [73]	EU	53	179	Longitudinal	5 to 16	BOP + PD ≥ 5 mm + BL > 0.2 mm/year	32.0755	23.4637

**Table 9:** Characteristics of studies Group 4 (other criteria)

Studies	Characteristics						Results	
	Population	Sample (N)(P)	Sample (N)(DI)	Design follow-up	Time load (Y)	Diagnostic	Rate (P) (%)	Rate (DI) (%)
Aguirre-Zorzamo [24]	EU	239	786	Cross-sectional	5.25 ± 3.4	BOP/sup + incre PD + BL ≥ 1.5 mm	15.0637	9.7964
Mameno [48]	Japan	477	1420	Cohort	5 to 10	BOP/sup + BL ≥ 1 mm	15.3040	9.2253
Menini [51]	EU	72	331	Longitudinal	5.8	BOP/sup + BL	6.9444	1.5106
Mir-Mari [52]	EU	245	964	Cross-sectional	6.3 ± 4.3	BOP/sup + BL ≥ 2thread	16.3265	9.1286
Renvert [60]	EU	213	976	Cross-sectional	10.8 ± 1.5	BOP/sup + BL > 3 exposed threads	15.0235	Missing
Roos-Jansàker [69]	EU	216	987	Cross-sectional	9 to14	BOP/sup + BL > 1.8 mm	16.2037	6.5856
Serino [70]	EU	23	109	Cross-sectional	5 to 10	BOP/sup + PD ≥ 6 mm	100	53.2110
Van Velzen [78]	EU	169	356	Cohort	10	BOP + BL ≥ 1.5 mm	14.7929	7.0225
Wada [79]	Japan	543	1613	Longitudinal	5.8 ± 2.5	BOP/sup + BL > 1 mm	15.8379	9.2374

The peri-implantitis mean prevalence obtained was 19.53% (95% CI, 12.87 to 26.19%) at the patient-level and 12.53% (11.67 to 13.39%) at the implant-level. Table 10 shows all the results of the study. Given the high specific weight of group 2 compared to the other groups (53.45%), the total results were calculated by weighted average.

**Table 10:** Means of peri-implantitis prevalence (%), with confidence interval (CI-95%) in parenthesis

Group	Patient-level	Implant-level
1	16.4 (0.9–31.89)	12.12 (2.96–21.29)
2	20.67 (15.89–25.44)	12.65 (8.98–16.31)
3	14.68 (4.13–25.23)	12.04 (4.71–19.37)
4	23.94 (1.91–45.98)	13.21 (0.45–25.98)
Total	19.53 (12.87–26.19)	12.53 (11.67–13.39)

In addition, an analysis of the influence of the load time or time variable was carried out based on implants on the registered peri-implantitis prevalence at the patient level and at the implant level, that is displayed in Table 11. No significant differences were observed in prevalence among studies with follow-up period of 5 to 9 years and studies with greater longevity, both at patient-level (17.1% vs. 18.63%, p = 0.82) as at implant-level (10.98% vs. 9.76%, p = 0.8).

**Table 11:** Prevalence of peri-implantitis (%) at patient-level and implant-level, in function of load time (CI-95%)

Group	Patient-level		Implant-level	
	5–9 y	> 9 y	5–9 y	> 9 y
**1**	14.18	17.13 (− 12.15 to 46.4)	21.3 (104.67 to 147.28)	8.45 (1.51–15.39)
**2**	20.57 (14.88 to 26.29)	21.2 (14.65 to 27.74)	12.32 (8.53 to 16.11)	8.78 (2.49–15,08)
**3**	12.11 (− 0.62 to 24.84)	15.04 (− 53.06 to83.14)	3.53 (− 4.2 to 11.25)	15.78 (12.07–19.48)
**4**	13.54 (6.49 to 20.59)	15.34 (13.46 to 17.21)	7.42 (1.13 to 13.7)	6.8 (4–9.6)
Total	17.1	18.63	10.98	9.76

Considering the Consensus report of the 2017 World Workshop on the classification of periodontal and peri-implant diseases and conditions [15] recommending that probing depth should not be included as a diagnostic criterion, studies have been divided according to this variable to study its impact on peri-implantitis prevalence (Table 12). Prevalence in studies that used probing depth, as one more diagnostic criterion was higher than those that did not used it, both at patient-level (24.69% and 17.56% respectively) and at implant-level (15.21% and 11.99% respectively). However, no significant differences were observed (p = 0.27 and p = 0.31 respectively).

**Table 12:** Peri-implantitis prevalence with/without probing depth inclusion (CI-95%)

PI with probing depth			PI without probing depth		
Group	Patient-level	Implant-level	Group	Patient-level	Implant-level
1	17.13 (− 12.1 to 46.41)	12.25 (0.86 to 23.62)	1	14.18	11.39
2	19.89 (14.09 to 25.69)	12.78 (7.24 to 18.08)	2	22.05 (11.85–32.24)	13.07 (7.87–17.34)
3	16.15 (5.03 to 25.75)	10.99 (1.82 to 21.95)	3	9.68	13.29 (8.4–18.18)
4	57.53(− 482.1 to 597.16)	31.05 (− 244.3 to 307.3)	4	14.35 (11.27–17.41)	7.12 (2.68–9.61)
Total	24.69	15.21	Total	17.56	11.99

**Fig. 2:**
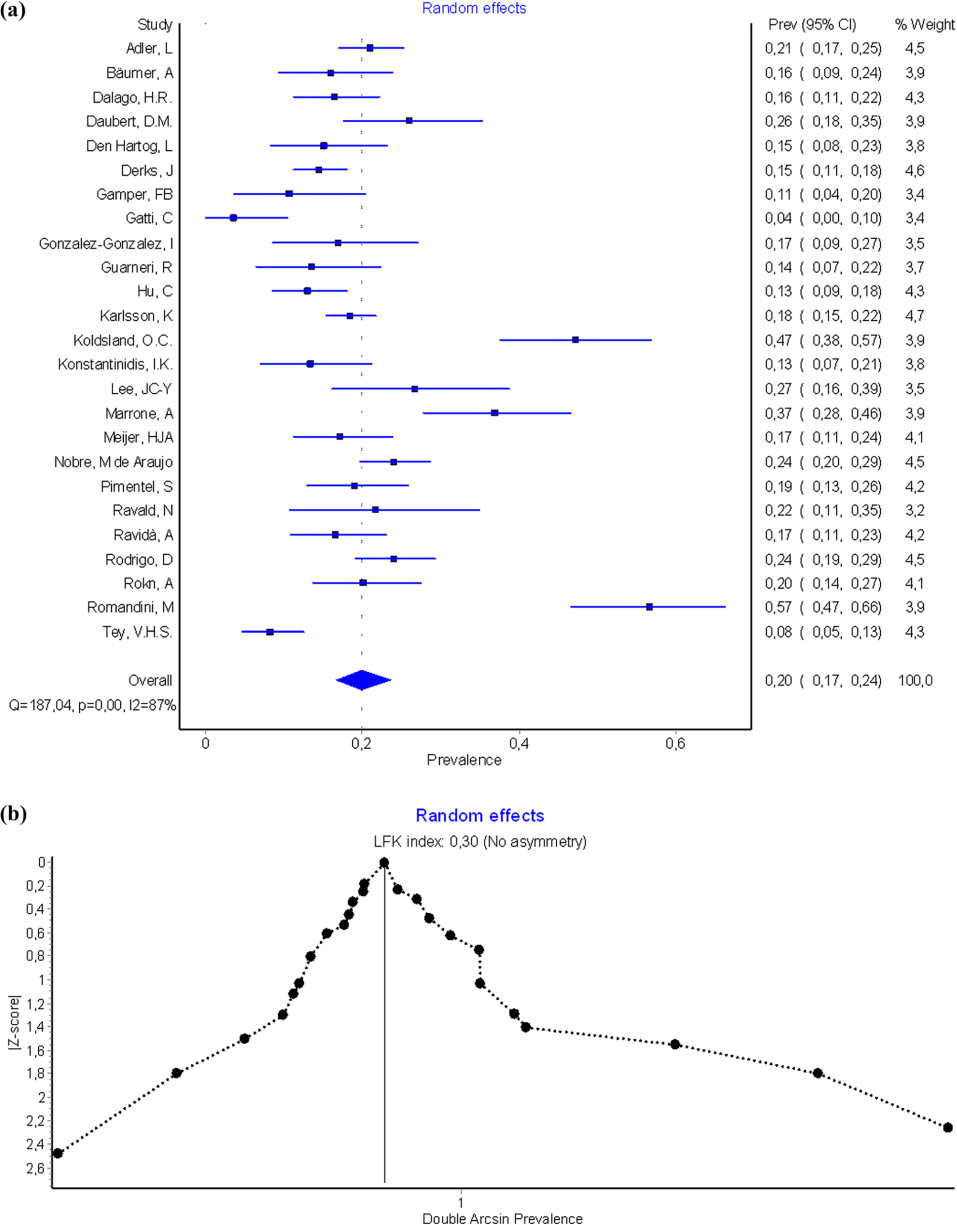

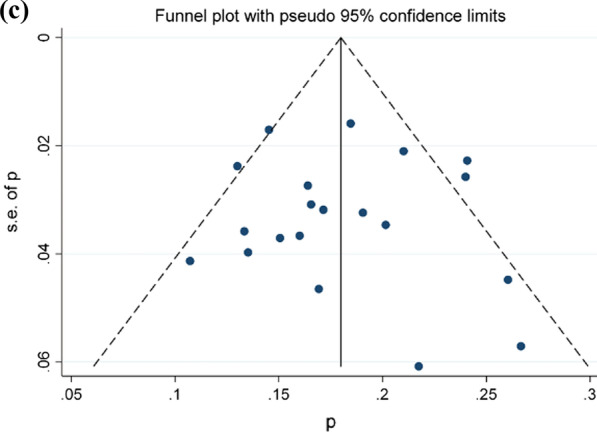
**a** Forest plot prevalence of peri-implantitis at patient-level. **b** Doi plot prevalence peri-implantitis at patient-level and LFK index analysis of publication bias. **c** Funnel plot prevalence of peri-implantitis at patient-level

The results of meta-analysis indicated a prevalence of peri-implantitis at patient level of 19.6% (CI-95%, 18.4–20.8) for the fixed effects model and 20% (CI-95%, 16.6–23.7) for the random effects model. Results of meta-analysis for the quality effects by income (studies with high effect size -Pimentel [56], Ravald [57], Rokn [67]—versus low/middle effects size -Romandini [68], Konstantinidis [46], Tey [75] -), were 19.2% (CI-95%, 15.2–23.6). Heterogeneity analysis in both models was high (I^2^ = 87.169%). (Fig. 2a, b shows Forest plot and Doi plot-LFK index). After performing the sensitivity analysis excluding studies of Gatti [39], Koldsland [45], Marrone [49], Romandini [68], and Tey [75] (Fig. 2c), the prevalence at the patient level for the random effects was 18.1% (CI-95%, 16.2–19.9%), with moderate heterogeneity (I^2^ = 44.3%). The Egger’s intercept test was 0.17 (CI-95%, 0.12–0.23; t = 6.70; df = 19; p = 0.846) and LFK index was 0.30 indicating no small-study effects.

**Fig. 3:**
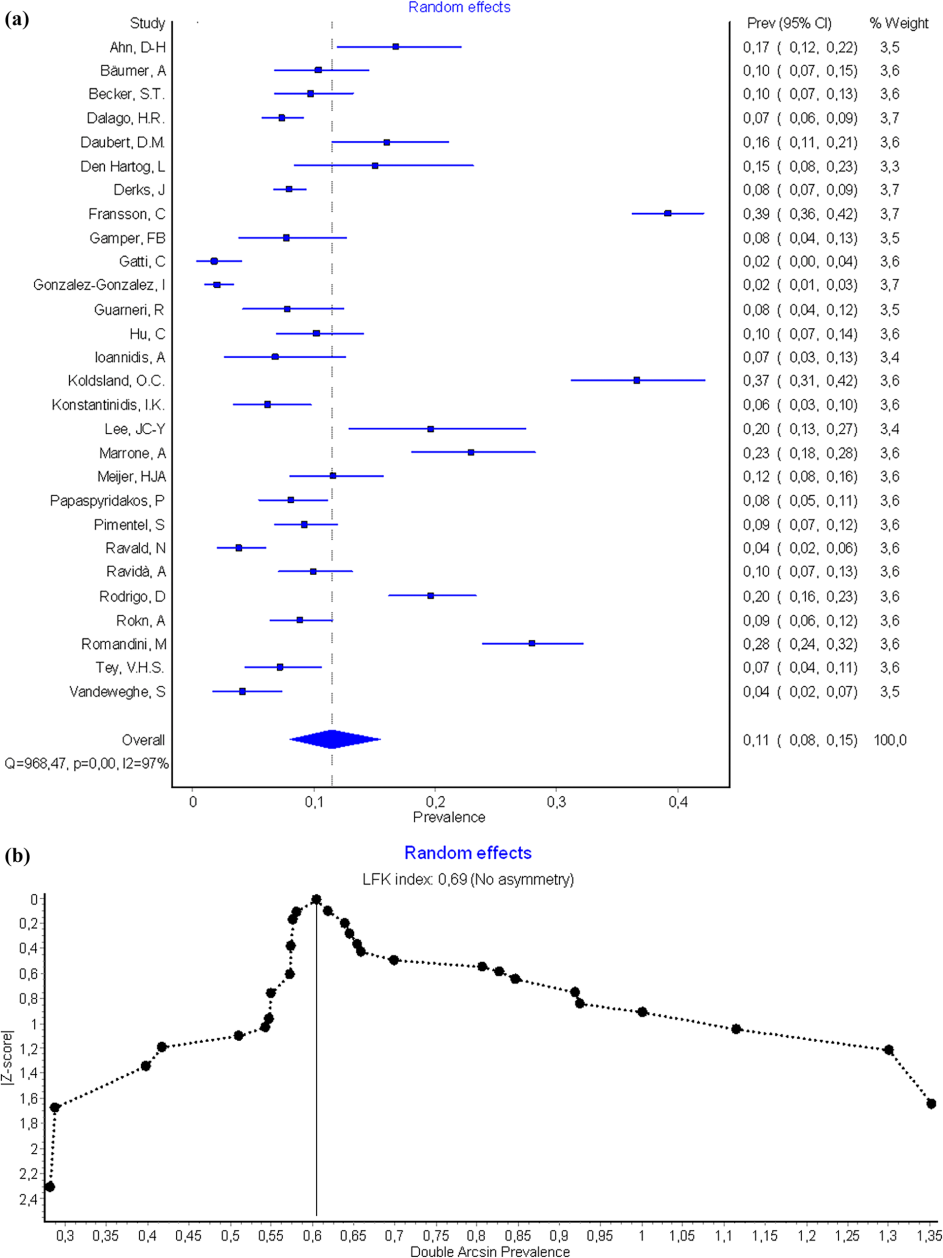

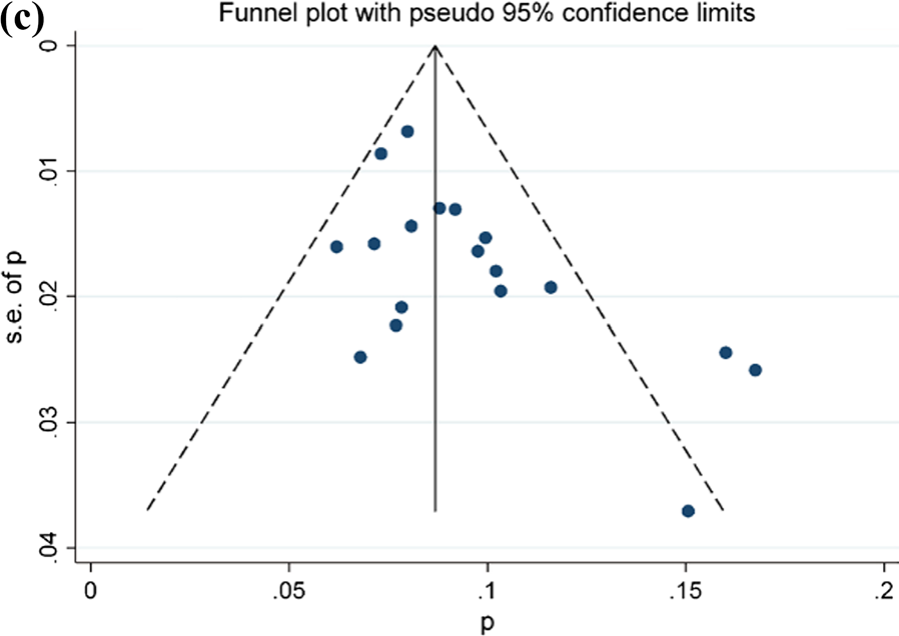
**a** Forest plot prevalence of peri-implantitis at implant-level. **b** Doi plot prevalence of peri-implantitis at implant-level and LFK index analysis of publication bias. **c** Funnel plot prevalence of peri-implantitis at implant-level

The prevalence of peri-implantitis at implant-level obtained was 12.3% (CI-95%, 11.7–12.9%) for the fixed effects model and 11.5% (CI-95%, 8–15.4%) for the random effects model. Results of meta-analysis for the quality effects by income (studies with high effect size-Meijer [50], Den Hartog [33], Bäumer [26]- vs. low/middle effects size -Fransson [36], Gonzalez-Gonzalez [40], Koldsland [45]-), was 11.1% (CI-95%, 7–15.9). High heterogeneity in both models was also observed (I^2^ = 97,21%). (Fig. 3a, b shows Forest plot and Doi plot-LFK index). After performing the sensitivity analysis excluding studies of Fransson [36], Gatti [39], González-González [40], Koldsland [45], Lee [47], Marrone [49], Ravald [57], Rodrigo [66], Romandini [68] and Vandeweghe [77] (Fig. 3c), the prevalence at the implant level was 9.1% (95% CI, 8.1–10.2%). Meta-analysis found heterogeneity among the studies (I^2^ = 46.2%). The Egger’s test was 0.06 (CI-95%, 0.00–0.39; t = 5.96; df = 17; p = 0.01) and LFK index was 0.69 indicating the presence of small-study effects (Fig. 3a, b).

## Discussion

The present systematic review highlighted some limitations of the definition, severity, and prevalence of peri-implantitis. Peri-implant health can exist around implants with reduced bone support. Peri-implantitis occurring in sites with clinical signs of inflammation, bleeding on probing and/or suppuration, increased probing depths and/or recession of the mucosal margin in addition to radiographic bone loss [15].

The case definition of peri-implantitis is affected by the different criteria used to define a “case” in studies investigating the prevalence of peri-implant diseases [80]. Discordance in disease definition among published studies makes the prevalence range highly variable and illustrates the lack of consensus in research, making it difficult to globally estimate the real elementary epidemiological parameters such as prevalence [13, 81]. In fact, there is currently a difference in how the peri-implantitis is defined in daily clinical practice and in epidemiological studies. Zitzmann and Berglundh [2] suggested that epidemiological research on peri-implant diseases should report not only on the prevalence or incidence of such but also on extent and severity. To determine the prevalence and incidence of peri-implantitis correctly, more prospective studies with adequate sample size and sampling method would be needed. In addition, baseline radiographic and probing measurements before and after loading the implant supported prosthesis must be performed to establish a bone level reference of physiological remodeling. Currently not many studies of this type are available. Most of the studies included in this research provided data from convenience samples, and most data were cross-sectional or collected retrospectively, rather than using randomized samples, resulting in a potential selection bias.

In the present systematic review, because a direct comparison was not possible, the referent case definition of peri-implantitis was subdivided into 4 groups with various thresholds for bone loss or exposed implant threads, and values for included peri-implant pocket depths, because only a few study protocols have applied the new classification of periodontal diseases of World Workshop on the Classification of Periodontal and Peri-Implant Diseases and Conditions [15]. The design was thoroughly done to review the published literature and to retrieve as much data as possible from the filtered papers.

Revised studies reported a mean prevalence for peri-implantitis of 19.53% at patient-level and 12.53% at implant-level. The global values reported at patient-level were similar to those previously reported by Ting et al. [82] (18.8%), Atieh et al. [83] (18.8%), and Lee et al. [84] (19.83%). However, the same authors reported lower prevalence values than in the present review at implant-level (9.25–9.6%). The differences may be because a small-study effect was found in the present study. Derks and Tomasi [13] also showed similar results at patient-level (21.7%). Conversely, Salvi et al. [85] reported lower prevalence values both at patient-level (10.3%) and at implant-level (7.5%).

Follow-up time and the evaluation in a convenience population may have influenced the prevalence values since peri-implantitis represents rather a chronic form of disease implying time for the osseous destruction [86]. Analyzing the influence of the period of functional loading, the results showed no differences in prevalence among studies with a follow-up period of 5 to 9 years and studies over 9 years of function, both at patient-level (17.19 and 17.75% respectively) and at implant-level (11.11 and 9.43% respectively). Conversely, Derks and Tomasi [13] meta-regression showed a significant positive relationship between the prevalence of peri-implantitis and mean function time, in a follow-up period of 3 to 9 years. Consistent with the present systematic review, Dreyer et al. [87] have reported that there is not an increase in the prevalence of peri-implantitis at patient-level due to longer functional loading period.

The authors are unaware of previous studies analyzing the influence of probing depth measurement and how it affects the prevalence of peri-implantitis. In this systematic review it was observed that the prevalence of peri-implantitis was higher when probing depth was used as one of the diagnostic criteria, but without significant differences. Hence the controversy of changes in the definition of peri-implantitis.

The meta-analysis of the prevalence of peri-implantitis should be interpreted with caution, due to the high heterogeneity found in the group 2. Muñoz Giraldo et al. [88] reported a prevalence of peri-implantitis of 18% at patient-level similar to the results of the study (20%). However, their I^2^ index of 95.7% was higher than in the present study (87.169%). At implant-level, the present study reported a prevalence of peri-implantitis of 11.5% with I^2^ index of 97.21%, and Muñoz Giraldo et al. [88] obtained a prevalence of 10% consistent with this data, also with a high heterogeneity (I^2^ = 95.0%). The differences in heterogeneity observed between both systematic reviews and meta-analysis, may be due to the greater number of studies (33) included in this study for the group analyzed (probing depth ≥ 6 mm).

Limitations of the study included that the methodology used in data collection did not record the ethnic differences in the populations of the selected studies. But the main limitation of this study was the small number of articles found with original prevalence data of peri-implantitis (number of cases and number of patients/implants). The strength of this study is its novelty when analyzing the prevalence by four diagnostic criteria.

## Conclusions

Within the limitations of this study, it can be concluded that prevalence of peri-implantitis, using 4 different definitions, was found to be approximately 20% at patient-level and 11.5% at implant-level. The results indicate that the identification of the peri-implantitis diagnostic criteria is essential to achieve greater accuracy in the disease classification, and for correct estimation of the true prevalence value of peri-implantitis. Further studies should use more consistent periodontal measurements, and using only the definition proposed in the 2017 World Workshop on the Classification of Periodontal and Peri-Implant Diseases and Conditions.

## Data Availability

All data analyzed during this study are included in this manuscript.

## Abbreviations

CI: Confidence Interval
BOP: Bleeding on Probe
PD: Probing Depth
